# PRESIDENTIAL SYMPOSIUM: REIMAGINING AGING AND LONG-TERM CARE FOR HEALTH EQUITY DURING AND POST-COVID-19: A HEALTH SCIENCES FOCUS

**DOI:** 10.1093/geroni/igac059.739

**Published:** 2022-12-20

**Authors:** Kirsten Corazzini, Deb Bakerjian

## Abstract

In line with the theme of the conference, this symposium highlights exemplars of health sciences scholars who are identifying key issues and opportunities to re-imagine aging and long-term care, through a health equity lens, as highlighted by COVID-19. This lens is inclusive of the wide range of long-term care stakeholders whose well-being and health outcomes have been affected throughout the COVID-19 pandemic, including older adults, family caregivers, and the healthcare workforce. We focus in particular on vulnerable aging populations, such as older adults who are living with neurocognitive disorders, racial/ethnic minority older adults, and older adults receiving palliative care. Finally, we consider these issues at both individual and systems-levels. Our first presenter examines person and family engagement in assisted living for older adults living with ADRD and the impact of COVID-19. The second presenter provides insights into palliative care needs in nursing homes, and the implications for transition to an endemic. The third presenter examines long-term care needs in the community, with a focus on the consequences of unmet needs for racial/ethnic minority older adults. The fourth presenter highlights the role and capacity of the nurse practitioner in nursing homes during COVID. Our final presenters provide a systems-level look at the COVID-19 response, focusing on self-organizing community coalitions to support nursing homes. Presentations reveal how re-imagining aging and long-term care in health sciences, requires consideration of health inequities experienced throughout COVID-19, whether newly emerging inequities, or long-standing challenges and inequities that have been exacerbated by COVID-19.

